# Novel Cut-Off Values of Precordial Voltage Indexes for Light Chain Amyloidosis Cardiomyopathy in a Chinese Population

**DOI:** 10.3390/jcdd13010044

**Published:** 2026-01-13

**Authors:** Ruokai Pan, Shengsheng Zhuang, Zeyuan Wang, Xiaoyu Ren, Zhuang Tian, Shuyang Zhang

**Affiliations:** 1 Department of Ultrasound, State Key Laboratory of Complex Severe and Rare Diseases, Peking Union Medical College Hospital, Chinese Academy of Medical Sciences and Peking Union Medical College, Beijing 100730, China; panruokai_pumc@163.com; 2 Department of Cardiology, The Xiamen Cardiovascular Hospital of Xiamen University, Xiamen 361006, China; 18813062239@163.com; 3 Department of Cardiology, Peking Union Medical College Hospital, Chinese Academy of Medical Sciences and Peking Union Medical College, Beijing 100730, China; zeyuanw2021@163.com (Z.W.); m13021806696@163.com (X.R.); 4 Department of International Medical Service, Peking Union Medical College Hospital, Chinese Academy of Medical Sciences and Peking Union Medical College, Beijing 100730, China

**Keywords:** electrocardiograph, cardiac amyloidosis, diagnostic threshold, voltage-mass ratio

## Abstract

Low QRS voltage relative to left ventricle (LV) thickness is one of the red flag characteristics in the diagnosis of cardiac amyloidosis, and it can be measured by specific indexes. Few studies have clearly defined the diagnostic threshold of voltage indexes for light chain amyloidosis cardiomyopathy (AL-CA) patients and other patients with LV hypertrophy. This case–control study analyzed electrocardiograms and echocardiograms of patients with AL-CA, hypertrophic cardiomyopathy (HCM), and hypertension left ventricular hypertrophy (HTN-LVH) seen at a single university center from 2008 to 2022. Low QRS voltage and three different precordial voltage indexes were evaluated. Diagnostic thresholds for rule-in and rule-out were calculated for AL-CA against each control group. A single voltage–mass ratio based on cross-sectional area (CSA) exhibited most accurate diagnostic accuracy, and the value of ≤1.72 aids the rule-in of AL-CA against other causes of left ventricular hypertrophy, providing a positive predictive value (PPV) of 86% versus HCM and 75% versus HTN-LVH.

## 1. Introduction

Low QRS voltage relative to LV thickness is one of the red flag characteristics in the diagnosis of cardiac amyloidosis (CA) [1]. However, simple ECG voltage has been criticized for its low sensitivity; for instance, the sensitivity of traditional low voltage criteria for ATTR-CM and AL-CM ranges from 25% to 40% and 50%, respectively [2]. Although far from satisfactory, ECG voltages are still the most used clinical indexes due to their convenience and noninvasive nature.

Unlike common cardiomyopathies with left ventricular (LV) hypertrophy like hypertrophic cardiomyopathy (HCM), LV hypertrophy in CA patients is caused by the deposition of amyloid in the extracellular matrix, which leads to decreased myocardial density and manifests as low voltage on ECG. However, other factors such as cardiac morphology and body geometry affect the value of ECG voltage as well, which means low voltage alone is not specific enough for the diagnosis of CA [3,4]. The discordance between the QRS voltage on the electrocardiogram (ECG) and the left ventricular wall thickness (LVWT) on echocardiography (echo) has been recognized as a danger sign of amyloid cardiomyopathy [5,6,7]. By combining ECG voltage with echocardiographic parameters, several ECG voltage indexes play the clearer auxiliary diagnostic significance in clinical practice [8,9]. Currently, few studies have clearly defined the diagnostic threshold of voltage indexes for AL-CA patients and other patients with LV hypertrophy. We attempted to describe the voltage indexes for patients with AL-CA and other cardiomyopathies with LV hypertrophy and to determine a threshold with diagnostic significance.

## 2. Methods

### 2.1. Study Patients

In this retrospective case–control study, patients were recruited from the medical records retrieval system of Peking Union Medical College Hospital (PUMCH) by corresponding diagnoses, including cardiac amyloidosis, hypertrophic cardiomyopathy, left ventricular hypertrophy due to hypertension, from 2008 to 2022. Any patients suspected of having AL-CA presenting with monoclonal components in serum or urine in the first place must meet one of the following criteria: (1) biopsy-proven CA, or (2) in the absence of an endomyocardial biopsy, histological documentation of Congo red staining in at least 1 involved organ together with evidence of amyloid cardiomyopathy defined by echocardiography. Hypertrophic cardiomyopathy and hypertension left ventricular hypertrophy were chosen as disease controls due to their similar characteristics of left ventricular hypertrophy. Inpatients with a discharge diagnosis of hypertrophic cardiomyopathy or hypertension left ventricular hypertrophy during the same period were recruited. All patients with bundle branch block were excluded from the beginning. Healthy controls were recruited from the routine physical examination center of PUMCH from the period of 2016 to 2022. All recruited healthy controls were free of heart-relevant complaints or cardiac disease history.

### 2.2. Voltage Indexes Measurements

Three voltage indexes were included in this study: Sokolow–Lyon voltage and two types of voltage–mass ratio, which are the most frequently used voltage indexes clinically and relatively easy to calculate in daily medical diagnosis. The 12-lead electrocardiogram and echocardiogram at the time of the diagnosis of each participant were obtained from the medical records retrieval system. Sokolow–Lyon voltage was calculated by the sum of S wave in V1 plus R wave in V5 or V6. One method of calculating the voltage–mass ratio is the division of Sokolow–Lyon voltage by left ventricular mass index (LVMI); the other method is Sokolow–Lyon voltage divided by LV wall cross-sectional area (CSA) indexed to body surface area as defined by Carroll [8,9].

For electrocardiographic characteristics, low limb voltage was defined as a QRS amplitude ≤ 5 mm (0.5 mV) in all limb leads (I, II, III, aVR, aVL, aVF), low precordial voltage was defined as a QRS amplitude ≤ 10 mm (1.0 mV) in all precordial leads (V1–V6), and pseudoinfarction was defined as the presence of abnormal Q waves in the absence of corresponding clinical or imaging evidence of myocardial infarction.

Echocardiographic quantitative parameters were measured based on current international recommendations. Specifically, maximum LVWT was measured for IVS and posterior wall in the parasternal long-axis view with M-mode approach or linear measurement from two-dimensional echocardiographic images, whichever was higher. LVMI was calculated by the M-mode method and indexed to body surface area.

### 2.3. Statistical Analysis

Descriptive statistics between the study groups were calculated. Continuous variables were expressed as either mean ± standard deviation or median with interquartile range (IQR) [25°; 75°], depending on the results of normality test, and were analyzed by the Student’s t-test or Wilcoxon rank test for comparison between two groups, respectively. Differences between groups were evaluated using the Mann–Whitney U test for continuous variables and the Chi-square (χ^2^) or Fisher’s exact test for dichotomous variables, as appropriate. Receiver operator characteristic (ROC) curve analysis was performed to determine the accuracy of ECG/echo indexes in predicting the presence of AL-CA. To determine threshold values, for rule-in, a high specificity of 90% was required, and for rule-out, a high sensitivity of 90% was required. Additional positive and negative predictive values were calculated. Delong test was applied for the comparison of ROC curves for voltage indexes among groups. The best diagnostic performance in detecting AL-CA was investigated according to the area under the curve (AUC). A *p*-value < 0.05 was considered statistically significant. All statistical analyses were performed using GraphPad Prism version 9.5.1 (GraphPad Software, LLC, 2022, Boston, MA 02110, USA) and MedCalc version 23.1.6 (MedCalc Software Ltd, Ostend, Belgium).

## 3. Results

### 3.1. Patient Characteristics

Among 1816 patients who were qualified for corresponding diagnosis, 1254 were accessible for ECG and echocardiogram analysis, and there were a total of 974 patients included in the final research, which included 325 AL-CA patients, 322 HCM patients, and 327 HTN-LVH patients. In addition, 149 healthy controls were also taken into analysis.

**Figure 1:**
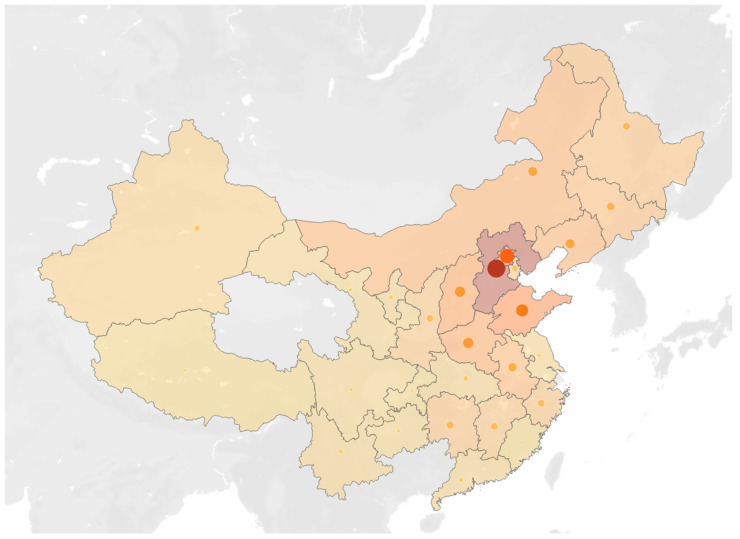
The distribution map of the registered permanent residence of all AL-CA patients. The darker the color and the larger the circle in each province, the greater the number of patients in that respective province.

The baseline characteristics of all four groups are shown in Table 1, while the native place map of CA patients is shown in Figure 1. AL-CA patients are quite similar with HCM patients in gender and age ratio, while HTN-LVH group shared more similar echocardiogram values such as LVPW and IVS with AL-CA patients. As for electrocardiograph characteristics, low voltage in either peripheral or chest leads, as well as pseudoinfarction patterns, were observed nearly only on AL-CA patients. In general aspects, compared with HCM and HTN-LVH, AL-CA patients possessed a lower ratio of hypertension and diabetes morbidity, ejection fraction, body surface area, left ventricle mass, but a higher LV diastolic dysfunction ratio.

Figure 1 indicates the distribution of the registered permanent residence of all AL-CA patients on the map of China. As a hospital located in northern China, most of the AL-CA patients attending PUMCH came from the North, Northeast, and Northwest regions of China, especially Beijing and the surrounding Hebei Province. However, as shown in the map, the AL-CA patients included in this study cover almost all provinces in China. A considerable number of patients were also included in this study from the provinces of South China, including Hunan, Jiangxi, and Zhejiang.

### 3.2. Voltage Indexes of Discordance

**Figure 2:**
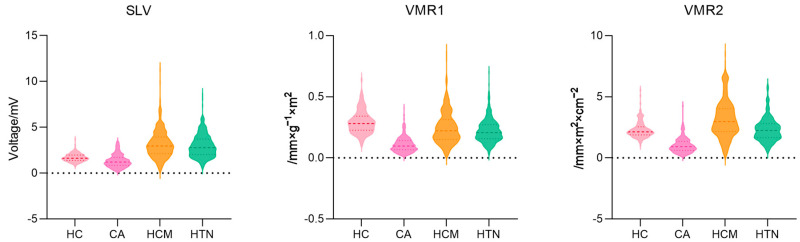
Three voltage indexes among four groups in the violin plot. SLV: Sokolow–Lyon voltage, VMR1: voltage–mass ratio calculated by left ventricular mass index, and VMR2: voltage–mass ratio calculated by cross-sectional area.

Table 2 and Figure 2 illustrate the median and distribution of three voltage indexes among the four groups. CA patients expressed consistently low value on all three voltage indexes with significant difference. HCM patients expressed the highest voltage indexes of all three kinds among the patient groups.

### 3.3. Diagnostic Test of Three Voltage Indexes

**Figure 3:**
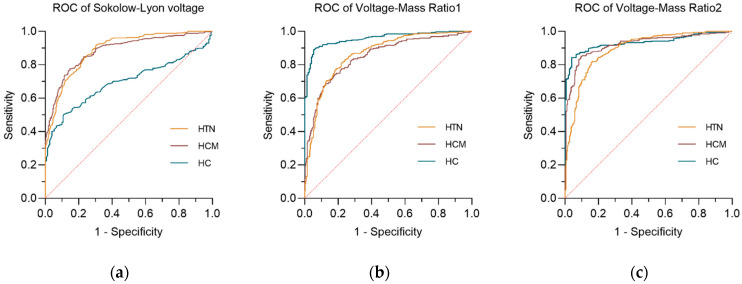
The receiver operating curve (ROC) of three voltage indexes in three comparative groups. (**a**) Sokolow–Lyon voltage; (**b**) voltage–mass ratio calculated by left ventricular mass index (VMR1); (**c**) voltage–mass ratio calculated by cross-sectional area (VMR2). The diagonal reference line (dashed) represents the performance of a test with no discriminative ability, serving as a baseline for comparison.

**Figure 4:**
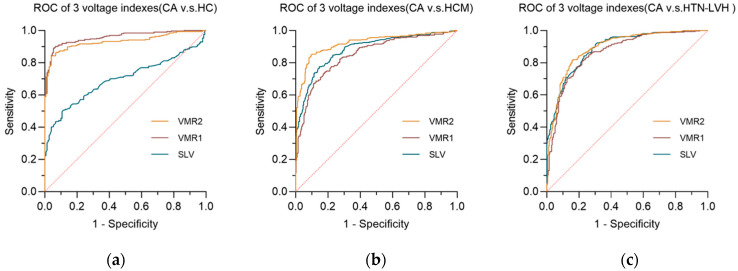
The receiver operating curve (ROC) of three voltage indexes in each comparative groups. (**a**) AL-CA *vs.* HC comparative group; (**b**) AL-CA *vs.* HCM comparative group; (**c**) AL-CA vs. HTN-LVH comparative group. SLV: Sokolow–Lyon voltage, VMR1: voltage–mass ratio calculated by left ventricular mass index, and VMR2: voltage–mass ratio calculated by cross-sectional area. The diagonal reference line (dashed) represents the performance of a test with no discriminative ability, serving as a baseline for comparison.

Table 3 summarizes the diagnostic performance of three voltage indexes upon three comparative groups by receiver operating characteristic curve (ROC). In the AL-CA *vs.* HCM group and the AL-CA *vs.* HTN-LVH group, the voltage–mass ratio/CSA (VMR2) both exhibited the highest AUC values of 0.9207 and 0.8927, respectively. Figure 3 and Figure 4 respectively describes the comparisons of the three voltage indexes in different patients and the comparisons of the three voltage indexes in the three comparison groups.

In the comparison of the three ECG voltage indexes within each of the three comparison groups, Sokolow–Lyon voltage (SLV) showed its poor diagnostic performance in AL-CA *vs.* HC, while the other two ECG voltage indexes performed better in the AL-CA *vs.* HC comparison group than in the other two comparison groups. In the comparison group of AL-CA patients and other left ventricular hypertrophy diseases, the voltage–mass ratio/CSA (VMR2) exhibited better diagnostic accuracy than the voltage–mass ratio/LVMI (VMR1) with significance (*p* < 0.05, DeLong’s test).

### 3.4. Diagnostic Thresholds for Different Voltage Indexes

As shown in Table 3, among all the indicators, VMR2 performed the best. Table 4 summarizes the cut-offs for three voltage indexes in three comparative groups. For rule-in purposes, the optimal cut-off points for SLV, VMR1, and VMR2 were calculated and compared as to their PPVs. Applying the rule-in cut-off of VMR2 ≤ 1.72, 85.4% of AL-CA patients were correctly identified against HCM and 70.6% against HTN-LVH. At this value, 10% or less of the non-CA population was also ruled-in, as defined by the requested specificity.

In order to simulate the identification of AL-CA in clinical practice, that is, distinguishing AL-CA from all other causes of left ventricular hypertrophy, we combined patients with HCM and HTN-LVH into the [HCM+HTN-LVH] group for comprehensive analysis. In the analysis of this combined group, when the criterion of specificity > 90% was set for the diagnosis of AL-CA, the cut-off values of Sokolow–Lyon voltage, VMR1, and VMR2 were 2.36 mV, 0.1824, and 1.738, respectively. The rule-in values are similar when compared with the HCM or HTN-LVH groups, respectively, indicating that indicators such as VMR2 can still provide stable and highly specific diagnostic cut-off values in a wider range of scenarios for the differential diagnosis of left ventricular hypertrophy.

When the standard with a sensitivity > 90% was set to exclude AL-CA, the corresponding critical values were 1.56 mV, 0.1096, and 1.300, respectively, in the analysis of the combined group. The best NPV was seen for SLV and VMR2 as 85.59% and 86.90%, respectively. However, for both indexes, the specificity was only around 70%, making both indexes less useful. Indeed, this indicates that 30% of non-AL-CA patients cannot be ruled out of possibly having AL-CA. For VMR2 specifically, when analyzed against each hypertrophy cohort separately, the cut-off value was ≥1.399 when compared to HCM alone, excluding 76.31% of the HCM patients, and ≥1.251 when compared to HTN-LVH alone, excluding 70.15% of HTN-LVH patients.

Referring to Carroll’s research for rule-out comparison, we further explored the lower critical value of VMR2 ≥ 1.175 [8]. At this critical value, in our population, the sensitivity decreased to 68.00%, the specificity was 91.68%, and the negative predictive value increased to 85.14%.

## 4. Discussion

This is the first large-scale study to conduct diagnostic tests on the precordial ECG voltage index of the Chinese AL-CA population with other left ventricular hypertrophy populations. This study evaluated the diagnostic accuracy of the three most commonly used and most convenient to calculate ECG voltage indexes in clinical practice. The main findings are as follows:

1. In the differential diagnosis of AL-CA patients from patients with hypertrophic cardiomyopathy and HTN-LVH, the voltage–mass ratio based on CSA exhibited better diagnostic accuracy compared to the other indexes;

2. The use of voltage–mass ratio/CSA of ≤1.72 as the rule-in threshold for AL-CA has good specificity of 90% and a PPV between 75% and 86% when compared to HTN-LVH and HCM, respectively;

3. Using a voltage–mass ratio/CSA ≥ 1.300 as the rule-out diagnostic threshold has good sensitivity but a limited specificity of 70%, which limits its possible use.

### 4.1. From Low QRS Voltage to Voltage–Mass Ratio in AL-CA Patients

Low voltage is not only a diagnostic feature of CA, but also listed as an important factor in the CA diagnostic prediction model by many studies; furthermore, it could act as a prognostic factor, which is relevant to poor prognosis [10,11]. The combination of ECG and echocardiography can not only help clinicians increase their suspicion of AL-CA and conduct subsequent diagnostic tests to confirm the presence of the disease (i.e., bone tracer scintigraphy, serum/urine immunofixation, and cardiac magnetic resonance), but also act the potential role as a predictor of adverse outcomes.

More than 30 electrocardiographic indexes for the diagnosis of left ventricular hypertrophy have been reported from the previous literature; however, the diagnosis is still generally based on the QRS voltage in clinical practice for ruling out, which results in a low diagnostic rate [12,13]. A recent study in LVH patients revealed that the ratio between the total QRS score and the maximum LVWT is the most accurate ECG/echo index, which suggests the diagnostic threshold of this index could have better clinical significance [7]. However, this index still appears rather complex in clinical practice. The balance between adopting simpler voltage indexes and achieving more accurate clinical diagnosis remains a clinical issue that needs to be addressed

### 4.2. Ruling in AL-CA and Voltage–Mass Ratio/CSA in Chinese Population

For the purpose of ruling in AL-CA within a Chinese population presenting with left ventricular hypertrophy, a VMR2 cut-off of ≤1.72 demonstrated high specificity (>90%) and substantial PPV (86% *vs.* HCM, 75% *vs.* HTN-LVH). It ensures that a positive test result carries a high degree of certainty, thereby justifying the initiation of more definitive but often invasive diagnostic procedures, such as tissue biopsy or scintigraphy, in patients flagged positive. The effectiveness of this rule-in strategy, however, is inherently dependent on the prior probability of AL-CA in the individual patient. Applying this high-specificity cut-off in a low-prevalence population would yield a lower positive predictive value than observed in our high-risk cohort, potentially increasing false positives. Conversely, its utility is maximized in a targeted population where clinical suspicion is already intermediate to high. Therefore, this tool is best employed to confirm diagnosis in patients with a suggestive clinical profile, rather than as a universal screening test.

Compared with Pagura’s study in Italy, Chinese patients with AL-CA in this study showed relatively higher VMR2 values (0.92, CI 0.62–1.36 *vs.* 0.83, CI 0.58–1.08), suggesting that the voltage indexes of CA patients may be affected by racial or regional factors [7]. We observed that among the 55 CA patients in the Italian cohort, 46 were ATTR-CA patients, which might explain the difference in VMR2 values between the two groups. Previous studies have consistently observed that AL-CA patients exhibit a lower voltage-to-mass ratio compared to those with ATTR-CA, with a higher proportion of AL-CA patients presenting a low ratio [14,15,16]. The higher mean voltage–mass ratio in ATTR-CA patients is consistent with the lower prevalence of short QRS complexes in ATTR-CA patients [17]. Furthermore, their non-AL group likely had comparatively lower VMR2 values. In contrast, our study specifically only included patients with AL-CA, and our Chinese control cohort demonstrated inherently higher VMR2 values.

To achieve the desired >90% sensitivity for rule-out in our population, a higher VMR2 cut-off of ≥1.30 was necessitated to make a better rule-out distinction between AL-CA and non-CA patients, since non-CA patients in our research have higher VMR2 values than Pagura’s population of non-CA. Even then, the specificity of 1.175 or 1.3 is not optimal in either case, so neither of them were not quite well to apply.

### 4.3. Rule-Out Considerations

Carroll’s research confirmed that the voltage–mass ratio/CSA is a good indicator for diagnosing CA, and a cut-off value of 1.175 was proposed [8]. Pagura’s study in Italy adopted this value and performed a sensitivity of 88.6% and a specificity of 74.2% in the cohort [7]. However, when we verified this critical value in this study cohort [AL-CA *vs.* (HCM+HTN)], its sensitivity was only 68.00%, and the negative predictive value (NPV) was 85.14%. This sensitivity is far from meeting the diagnostic criterion of reliable exclusion, meaning that if this criterion is applied, approximately 32% of AL-CA patients will be wrongly classified into the non-AL-CA group. In contrast, the rule-out cut-off value (VMR2 ≥ 1.300) in this study determined based on the 90% sensitivity criterion in this study performed better with a specificity of 70.15% and an NPV of 86.9%; despite this, its specificity is still not ideal, as approximately 30% of non-AL-CA patients will still be misjudged as positive. The difference in this value between this study and the literature (1.30 *vs.* 1.175) can be attributed to the difference in the subgroup composition of CA patients as stated in Section 4.2, while the composition of population and the patient’s disease course may also interfere. The cohort of this study included a relatively large number of patients with advanced AL-CA (among the 256 patients with known Mayo stages, 96.1% were at stage II/III), which might also lead to some bias.

In conclusion, we cannot recommend the VMR2 ≥ 1.30 as the rule-out threshold in this study. Our results also indicate that critical values based on Western populations (such as 1.175) may lead to inaccurate results in other regions of the world. Future research should further optimize this critical value in multi-center prospective cohorts to balance high sensitivity with more acceptable specificity.

### 4.4. Limitations

Firstly, we used M-mode echocardiography to calculate the left ventricular mass. Compared with other more precise methods (such as cardiac magnetic resonance or three-dimensional echocardiography), this technique tends to overestimate the actual mass [18]. However, we chose this method based on the following considerations: (1) This study is a retrospective analysis. M-mode is the most widely used and accessible standardized measurement method in clinical practice, which ensures that we can systematically obtain data from a large number of historical medical records; (2) the main objective of this study is not to precisely report the absolute cardiac mass value, but to explore the diagnostic performance of the ratio indicators derived from this mass. Although M-mode may lead to systematic overestimation, this bias may be consistently present in all study groups (AL-CA, HCM, HTN-LVH). Future prospective studies that verify using superior techniques such as three-dimensional ultrasound or cardiac magnetic resonance will be of great value.

In addition, this study was a single-center retrospective study, which may introduce selection bias and limit generalizability. The included patients did not cover the full spectrum of disease progression, and the charts did not provide enough information to indicate the NYHA class of the patients when CA was confirmed by myocardial biopsy. It has been shown that clinical heart failure is closely associated with the degree of echocardiographic abnormalities, especially increased wall thickness [19]. A significant proportion of CA patients are misdiagnosed with hypertrophic cardiomyopathy [11]. Since not all patients in the HCM group underwent cardiac biopsy to exclude the possibility of CA, some CA patients may have been included in the HCM group, resulting in bias. Baseline demographic data were limited to data recorded in the medical records, and some patients with missing data were excluded from the study. Most of the patients lived in northern China, where differences in climate, dietary habits, and cardiovascular disease prevalence—such as greater sodium intake and a relatively higher rate of hypertension—may influence the accuracy of the diagnostic test. Furthermore, external validation of all threshold cut-offs is warranted.

## 5. Conclusions

The voltage–mass ratio based on CSA is most suitable for the differential diagnosis of CA patients from patients with hypertrophic cardiomyopathy and HTN-LVH. Using this index, a rule-in cut-off of 1.72 can be used as the diagnostic threshold for AL-CA *vs.* HTN-LVH and HCM.

## Figures and Tables

**Table 1:** Baseline clinical characteristics.

	AL-CA (n = 325)	HCM (n = 322)	HTN-LVH (n = 327)	HC (n = 149)	*p*-Value
Age, years	60 (51–66)	62 (51–69) *	47 (34–60) **	47 (41–54) ***	<0.0001
Female, %	116 (35.7)	134 (41.6)	74 (22.6) **	62 (41.6) **	<0.0001
Height, cm	168 (160–172)	168 (160–173)	171 (165–177) **	169 (163–175)	<0.0001
Weight, kg	62 (55–70)	69 (60–80) *	77 (67–88) **	69 (62–79) ***	<0.0001
Hypertension, %	67 (20.6)	151 (46.9) *	327 (100%) **	20 (13.4)	<0.0001
Diabetes, %	38 (11.7)	72 (22.4) *	90 (27.5) **	5 (0) ***	<0.0001
Low limb voltage, %	97 (29.8)	1 (0) *	0 (0) **	0 (0) ***	<0.0001
Low precordial voltage, %	80 (24.6)	0 (0) *	0 (0) **	0 (0) ***	<0.0001
Pseudoinfarction, %	34 (10.5)	0 (0) *	0 (0) **	0 (0) ***	<0.0001
LVEDD, mm	43 (40–47)	47 (43–50) *	51 (47–54) **	46 (44–49) ***	<0.0001
LVPW, mm	13 (11–14)	10 (8–11) *	12 (11–13) **	8 (7–8) ***	<0.0001
IVS, mm	14 (12–15)	16 (13–18) *	12 (12–13) **	8 (7–9) ***	<0.0001
EF%	59 (51–68)	69 (64–73) *	64 (59–70) **	66 (63–70) ***	<0.0001
E/A < 1 or E/A > 2	206 (63.4)	186 (57.8)	173 (52.9) **	29 (19.5) ***	<0.0001
Body Surface Area, m^2^ #	1.67 ± 0.18	1.74 ± 0.22 *	1.87 ± 0.25 **	1.76 ± 0.21 ***	<0.0001
LVM, g	213 (171–264)	232 (181–279) *	249 (207–298) **	114 (99, 136) ***	<0.0001
CSA, cm^2^/m^2^	22.9 (11.6–14.4)	17.3 (14.3–21.1) *	23.8 (20.7–27.0) **	13.1 (11.6–14.4) **	<0.0001
LVMI, g/m^2^	129 (103–153)	131 (108–162) *	133 (113–157) **	66 (57–75) ***	<0.0001

Continuous variables are expressed as median (25% percentile-75% percentile), except where indicated. #: data are mean ± S.D. Categorical variables are presented as n (%). *p*-value indicates differences across four groups. *, **, and *** indicate significant differences (*p*-value < 0.05) between CA group and HCM group, CA group and HTN-LVH group, and CA group and HC group, respectively.

**Table 2:** Median value of three voltage indexes among four groups.

	AL-CA (n = 325)	HCM (n = 322)	HTN-LVH (n = 327)	HC (n = 149)	*p*-Value
Sokolow–Lyon voltage	1.20 (0.85–1.72)	2.98 (2.15–3.94) *	2.77 (2.02–3.73) **	1.63 (1.40–1.98) ***	<0.0001
Voltage–mass ratio/LVMI	0.097 (0.067–0.142)	0.224 (0.153–0.314) *	0.207 (0.158–0.272) **	0.280 (0.227–0.342) ***	<0.0001
Voltage–mass ratio/CSA	0.92 (0.62–1.36)	2.96 (2.16–4.04) *	2.24 (1.65–2.80) **	2.14 (1.88–2.54) ***	<0.0001

Continuous variables are expressed as median (25% percentile-75% percentile). *p*-value indicates differences across four groups. *, **, and *** indicate significant difference (*p*-value < 0.05) between CA group and HCM group, CA group and HTN-LVH group, and CA group and HC group, respectively.

**Table 3:** Diagnostic performance of three ECG/echo indexes for the identification of AL-CA with healthy control and other LV hypertrophy cardiomyopathies.

Group/*AUC*	Sokolow–Lyon Voltage (mV)	Voltage–Mass Ratio/LVMI (mm × g^−1^ × m^2^)	Voltage–Mass Ratio/CSA (mm × m^2^ × cm^−2^)
AL-CA versus HC	0.6962 ^A,B,a,b^ (0.6503–0.7421)	0.9575 ^A,C,a,b^ (0.9409–0.9741)	0.9324 ^B,C,a,b^ (0.9101–0.9547)
AL-CA versus HCM	0.8834 ^A,B,a^ (0.8575–0.9094)	0.8499 ^A,C,a^ (0.8202–0.8795)	0.9207 ^B,C,a,c^ (0.8986–0.9428)
AL-CA versus HTN-LVH	0.8888 ^A,b^ (0.8646–0.9131)	0.8646 ^A,C,b^ (0.8368–0.8925)	0.8927 ^C,b,c^ (0.8682–0.9173)

^A^, ^B^, and ^C^ indicate significant difference (*p*-value < 0.05 in DeLong’s test) in one group between SLV and VMR1, SLV and VMR2, and VMR1 and VMR2, respectively; ^a^, ^b^, and ^c^ indicate significant difference (*p*-value < 0.05 in DeLong’s test) in one voltage index between HC and HCM, HC and HTN-LVH, and HCM and HTN-LVH, respectively. SLV: Sokolow–Lyon voltage, VMR1: voltage–mass ratio calculated by LVMI, and VMR2: voltage–mass ratio calculated by CSA.

**Table 4:** Cut-off values for three voltage indexes in three comparative groups.

	Sokolow–Lyon Voltage (mV)	Voltage–Mass Ratio/LVMI (mm × kg^−1^ × m^2^)	Voltage–Mass Ratio/CSA (mm × m^2^ × cm^−2^)
**AL-CA versus HCM**			
cut-off value at 90% sensitivity	1.54	0.1069	1.399
specificity% at 90% sensitivity	68.92	57.23	76.31
NPV% at 90% sensitivity	74.29	67.60	79.02
cut-off value at 90% specificity	2.30	0.1811	1.723
sensitivity% at 90% specificity	69.57	63.66	85.40
PPV% at 90% specificity	74.68	71.46	86.14
**AL-CA versus HTN-LVH**			
cut-off value at 90% sensitivity%	1.58	0.1180	1.251
specificity% at 90% sensitivity%	70.15	62.77	70.15
NPV% at 90% sensitivity	90.15	71.01	75.13
cut-off value at 90% specificity%	2.30	0.1811	1.717
sensitivity% at 90% specificity%	66.06	62.39	70.64
PPV% at 90% specificity	72.28	70.19	75.26
**AL-CA versus HCM+HTN-LVH**			
cut-off value at 90% sensitivity%	1.56	0.1096	1.300
specificity% at 90% sensitivity%	69.85	62.77	70.15
NPV% at 90% sensitivity	85.59	81.91	86.90
cut-off value at 90% specificity%	2.36	0.1824	1.738
sensitivity% at 90% specificity%	66.26	62.39	70.64
PPV% at 90% specificity	56.78	54.56	85.92

## Data Availability

The original contributions presented in this study are included in the article/supplementary material. Further inquiries can be directed to the corresponding authors.

## Supplementary Materials

The following supporting information can be downloaded at: https://www.mdpi.com/article/10.3390/jcdd13010044/s1, STROBE Statement—checklist of items that should be included in reports of observational studies.

## Abbreviations

The following abbreviations are used in this manuscript:

AUC	Area under curve
AL	Light chain amyloidosis
ATTR	Transthyretin amyloidosis
CA	Cardiac amyloidosis
CSA	Cross-sectional area
ECG	Electrocardiograph
EF	Ejection fraction
HCM	Hypertrophic cardiomyopathy
HTN-LVH	Hypertension left ventricular hypertrophy
IVS	Inter ventricular septum
LVEDD	Left ventricular end diastolic diameter
LVMI	Left ventricular mass index
LVPW	Left ventricular posterior wall
LVWT	Left ventricular wall thickness
NPV	Negative predictive value
PPV	Positive predictive value
ROC	Receiver operating characteristic
