# Supplementary material for: Novel Cut-Off Values of Precordial Voltage Indexes for Light Chain Amyloidosis Cardiomyopathy in a Chinese Population
Source: J Cardiovasc Dev Dis. 2026 Jan 13;13(1):44. doi: 10.3390/jcdd13010044 (PMC12841747; doi:10.3390/jcdd13010044)
Supplement: Supplementary file 1 [file jcdd-13-00044-s001.zip › jcdd-3915336-supplementary.pdf]

STROBE Statement—checklist of items that should be included in reports of observational studies

	Item No.	Recommendation	Page No.	Relevant text from manuscript
**Title and abstract**	1	(*a*) Indicate the study's design with a commonly used term in the title or the abstract	1	Abstract, line 20-22, "This case-control study …from 2008-2022."
		(*b*) Provide in the abstract an informative and balanced summary of what was done and what was found	1	Abstract, line 20-28, "This case-control study … than male."
**Introduction**				
Background/rationale	2	Explain the scientific background and rationale for the investigation being reported	1	Line 33-38, "Low QRS voltage … noninvasive nature."
Objectives	3	State specific objectives, including any prespecified hypotheses	1, 2	Line 39-52, "Unlike common cardiomyopathies …diagnostic significance."
**Methods**				
Study design	4	Present key elements of study design early in the paper	2	Line 54-101
Setting	5	Describe the setting, locations, and relevant dates, including periods of recruitment, exposure, follow-up, and data collection	2	Line 54-68, "In this retrospective case-control study…cardiac disease history."
Participants	6	(*a*) *Case-control study*—Give the eligibility criteria, and the sources and methods of case ascertainment and control selection. Give the rationale for the choice of cases and controls		Line 58-64, "Any patients who suspected AL-CA … ventricular hypertrophy."
		(*b*) *Case-control study*—For matched studies, give matching criteria and the number of controls per case	N/A	N/A
Variables	7	Clearly define all outcomes, exposures, predictors, potential confounders, and effect modifiers. Give diagnostic criteria, if applicable	N/A	N/A
Data sources/ measurement	8*	For each variable of interest, give sources of data and details of methods of assessment (measurement). Describe comparability of assessment methods if there is more than one group	*2*	*Line 69-83*
Bias	9	Describe any efforts to address potential sources of bias	N/A	N/A
Study size	10	Explain how the study size was arrived at	N/A	N/A

Quantitative variables	11	Explain how quantitative variables were handled in the analyses. If applicable, describe which groupings were chosen and why	2	Line 70-83
Statistical methods	12	(*a*) Describe all statistical methods, including those used to control for confounding	2,3	Line 85-101, “Descriptive statistics between ...”
		(*b*) Describe any methods used to examine subgroups and interactions	2,3	Line 85-101, “Descriptive statistics between ...”
		(*c*) Explain how missing data were addressed	N/A	N/A
		(*d*) *Case-control study*—If applicable, explain how matching of cases and controls was addressed	N/A	N/A
		(*e*) Describe any sensitivity analyses	2,3	Line 85-101, “Descriptive statistics between ...”
**Results**				
Participants	13*	(a) Report numbers of individuals at each stage of study—eg numbers potentially eligible, examined for eligibility, confirmed eligible, included in the study, completing follow-up, and analysed	3	Line 104-108, “Among 1816 patients …into analysis.”
		(b) Give reasons for non-participation at each stage	3	Line 104-108, “Among 1816 patients …into analysis.”
		(c) Consider use of a flow diagram	N/A	N/A
Descriptive data	14*	(a) Give characteristics of study participants (eg demographic, clinical, social) and information on exposures and potential confounders	3,4	Line 109-117, line 125-131
		(b) Indicate number of participants with missing data for each variable of interest	N/A	N/A
		(c) *Cohort study*—Summarise follow-up time (eg, average and total amount)	N/A	N/A
Outcome data	15*	*Cohort study*—Report numbers of outcome events or summary measures over time	N/A	N/A
		Case-control study—Report numbers in each exposure category, or summary measures of exposure		
		Cross-sectional study—Report numbers of outcome events or summary measures	N/A	N/A
Main results	16	(*a*) Give unadjusted estimates and, if applicable, confounder-adjusted estimates and their precision (eg, 95% confidence interval). Make clear which confounders were adjusted for and why they were included	3	Line 109-117
		(*b*) Report category boundaries when continuous variables were categorized	N/A	N/A
		(*c*) If relevant, consider translating estimates of relative risk into absolute risk for a meaningful time period	N/A	N/A

Continued on next page

Other analyses	17	Report other analyses done—eg analyses of subgroups and interactions, and sensitivity analyses	7, 8	Line 188-194, line 199-204
**Discussion**				
Key results	18	Summarise key results with reference to study objectives	9, 10	Line 223-234
Limitations	19	Discuss limitations of the study, taking into account sources of potential bias or imprecision. Discuss both direction and magnitude of any potential bias	10	Line 269-281
Interpretation	20	Give a cautious overall interpretation of results considering objectives, limitations, multiplicity of analyses, results from similar studies, and other relevant evidence	11	Line 283-288
Generalisability	21	Discuss the generalisability (external validity) of the study results	N/A	N/A
**Other information**				
Funding	22	Give the source of funding and the role of the funders for the present study and, if applicable, for the original study on which the present article is based	11	Line 297-300

*Give information separately for cases and controls in case-control studies and, if applicable, for exposed and unexposed groups in cohort and cross-sectional studies.

Note: An Explanation and Elaboration article discusses each checklist item and gives methodological background and published examples of transparent reporting. The STROBE checklist is best used in conjunction with this article (freely available on the Web sites of PLoS Medicine at http://www.plosmedicine.org/, Annals of Internal Medicine at http://www.annals.org/, and Epidemiology at http://www.epidem.com/). Information on the STROBE Initiative is available at www.strobe-statement.org.
